# Psychological impact of COVID-19 and lock down measures: An online cross-sectional multicounty study on Asian university students

**DOI:** 10.1371/journal.pone.0253059

**Published:** 2021-08-03

**Authors:** Karuthan Chinna, Sheela Sundarasen, Heba Bakr Khoshaim, Kamilah Kamaludin, Mohammad Nurunnabi, Gul Mohammad Baloch, Syed Far Abid Hossain, Areej Sukayt, Nevi Dalina, Usha Rajagopalan, Ramesh Kumar, Zahid Memon

**Affiliations:** 1 School of Medicine, Faculty of Health and Medical Sciences, Taylor’s University, Subang Jaya, Selangor, Malaysia; 2 Department of Accounting, Prince Sultan University, Riyadh, Saudi Arabia; 3 Deanship of Educational Services, Prince Sultan University, Riyadh, Saudi Arabia; 4 College of Business Administration, International University of Business Agriculture and Technology (IUBAT), Dhaka, Bangladesh; 5 Health Services Academy, Chak Shahzad, Islamabad, Pakistan; 6 Center of Excellence in Women and Child Health, Aga Khan University, Karachi, Pakistan; Columbia University, UNITED STATES

## Abstract

The COVID-19 pandemic and the lockdown measures have taken a toll on every level of the society, worldwide. This study examines their psychological impact on university students in Asia. A cross-sectional online survey was conducted between April and May 2020 in Malaysia, Saudi Arabia, Pakistan, Bangladesh, China, India and Indonesia. The Zung’s self-rating anxiety scale (SAS) and questions on adaptive and maladaptive coping strategies were used in this study. A total of 3,679 students from the seven countries participated in this study. Overall, 21.9% and 13.7% of the students in this study experienced mild to moderate and severe to extreme levels of anxiety. More than 20% of the students from China and Bangladesh reported severe to extreme level of anxiety compared to below 10% of the students from Indonesia, Malaysia and India. Among the female students, 15.9% experienced severe to extreme level of anxiety compared to 10.6% among the males. Females from Bangladesh, China, Malaysia, Pakistan and Saudi Arabia experienced significantly higher levels of anxiety compared to their male counterparts. Acceptance was the most used and Seeking Social Support was the least used coping strategies among the students. There were significant differences in the usage of the four strategies by countries. Stressors are predominantly financial constraints, remote online learning, and uncertainty related to their academic performance, and future career prospects.

## Introduction

COVID-19, the defining global health crisis of this millennium was first reported in Wuhan, China in December 2019. This highly infectious disease had spread to almost every country in the world, just within three months. At the time of writing, a total of 235 countries have been identified with COVID-19 cases, exceeding a total of 35 million people world-wide and a total death of more than one million. The cases are rising, and the infections are soaring again in some nations that have had obvious success in containing previous outbreaks. Governments across the world have been forced to limit public movement with extreme measures such as quarantines, lockdowns, social isolation, and restriction of movement in a bid to combat the spread of this deadly disease. The mitigation measures taken by the countries have resulted in huge financial loses in almost every industry, in every country and have also resulted in enormous psychological consequences [1–3]. As other public health emergencies, COVID-19 pandemic affect one’s psychological state in the short-term and mental health in the long-term [4].

**Fig 1:**
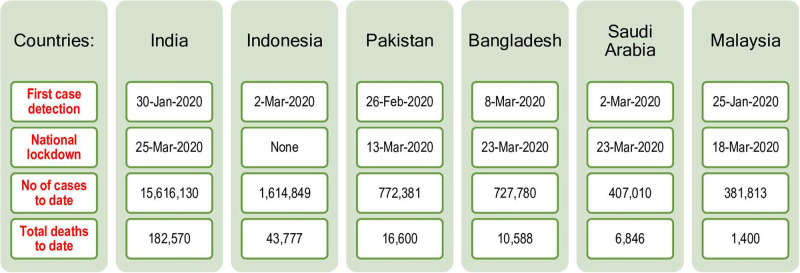
COVID-19 case detection, lockdown, number of cases and death.

Fig 1 details some background information about the first COVID-19 case detection, national lockdown imposition measure, total number of COVID-19 cases and fatalities in each country. Malaysia was the earliest in identifying and confirming COVID-19 case on its home ground followed by India in January 2020. With the exception of Indonesia, most countries started its national lockdown from mid- to end-of March 2020. Though widely criticised, Indonesian government only approved for large-scale social restrictions instead of imposing national lockdown. To date, India has the highest number of COVID-19 cases and fatalities, followed by Indonesia (worldometers.info).

As widely documented, during this pandemic, many academic institutions shifted from face-to-face to virtual mode of delivery. According to UNESCO, almost 600 million learners are affected world-wide due to the pandemic (UNESCO, 2020). This new norm of teaching and learning is very challenging for the students [5] and the teachers alike. It has caused tremendous adverse psychological outcomes, such as loneliness, worsening anxiety, distress and insomnia [6–9]. Graduating and/or final year students are overly concerned about their future, career, and further study plans amid this global health crisis [10].

Youths, specifically the students, are the most vulnerable group as they are stormed with numerous impetus that leads to depression and anxiety [11, 12]. Several studies conducted on mental health among university students have reported high prevalence of psychological symptoms [13–15]. According to Bao et al., [16], widespread outbreaks of an infectious disease, such as COVID-19, are associated with psychological distress and symptoms of mental illness. In a study conducted in China two weeks after the outbreak, it was found that 14.4% of the youth exhibited post-traumatic stress disorders [4]. Social isolation, anxiety, distress of contagion, ambiguity, chronic stress, and economic hitches could worsen stress-related conditions and attempts of suicide, mainly among those with pre-existing psychiatric disorders, low-resilient persons, those living in high COVID-19 occurrence areas, and individuals whose family member or friend who succumb to COVID-19 [17]. A survey conducted in a Spanish university found that 50.43% of the participants experienced moderate to severe psychological distress in the first week of their confinement [18]. Lower ability to record sensory input (hyposensitivity) is significantly correlated with higher levels of depression, which is mostly due to weakened ability to sense stimuli [19]. Similarly, Islam et al. [20] reported that around 15% of the students in Bangladesh had moderate to severe depression, whereas 18.1% were suffering from severe anxiety. The impact seems to be more severe among the older students who were self-financing their higher-level education.

## Coping mechanisms

Coping refers to the opinions and actions used by individuals to manage troubling situations. Coping mechanism involves repetitive mental and behavioural exertions to manage explicit external and/or internal difficulties that are considered as strenuous or beyond the resources of individuals [20]. Different people may use different coping strategies in dealing the same stressor. Adaptive coping strategies are largely characterised by individuals facing problems directly, ensuring rationally representative assessments of complications, identifying and altering unhealthy emotional responses. Adaptive coping strategies include problem-solving, seeking social and emotional support [21] that help to reduce stress, promote psychological well-being and improve general health outcomes [22].

Passive coping strategies generally involve avoidance, self-blaming, and substance use which are maladaptive in nature and are more prevalent among young adults [23, 24]. Avoidance is mainly because one’s thinking that he or she has little control to deal with the stressor [25]. Although avoidance may seem to dispel stress temporarily, the cumulative effect will become more complex at a later stage [26] and may lead to major and depressive symptoms [27] and anxiety [23]. People who use maladaptive coping strategies are known to have low life satisfaction, high negative thinking and they tend to exhibit a fight-flight response [28]. Sex, age, and level of study are known to be associated with maladaptive coping and anxiety amongst students [23, 29]. It is believed that more females use maladaptive coping strategies since they are more inclined to think negatively than males [30].

The purpose of this study is to examine and compare the socio-psychological wellbeing and the coping strategies used by university students during the period of COVID-19 pandemic in seven selected countries; India, Bangladesh, Saudi Arabia, Pakistan, Indonesia, China and Malaysia. These countries were chosen as they represent diversity in terms of total number of cases, socio-economic status and cultural. Inter-country factors that may have a socio-psychological impact of COVID-19 pandemic on students is investigated and compared as these countries have responded differently to the pandemic. This study also prescribes specific socio-psychological intervention strategies based on the outcome of the study. We believe this cross-country study is insightful to the understanding of anxiety and the coping strategies used by university students in different socio-cultural environments.

## Methods

### Ethical clearance, participants, procedure, and survey timeframe

This study was conducted simultaneously in the seven selected countries. Research ethics for this study was approved by the Prince Sultan University Institutional Review Board (PSU IRB-2020-04-0038). For this study, a questionnaire was designed and administered online using social media and emails. Data were collected by the country collaborators between April and May 2020. In the online survey, the respondents were briefed on the purpose of the study and written consents were taken from those willing to take part in the study. Assurance was given to participants that their involvement in the study was strictly voluntary and that their response would be anonymous.

### Research instrument

The questionnaires consisted of students’ general demographics, Zung’s self-rating anxiety scale (SAS) [31], and coping strategies used during the pandemic and restriction orders. In addition, the students were also asked to state their main concerns during this testing time.

### Zung’s self-rating anxiety scale (SAS)

Zung’s SAS [31] is used to assess the level of anxiety among the respondents. It is a validated 20-item self-report instrument that covers both psychological and somatic symptoms. In this instrument, items are scored on a scale of 1 to 4; “1 = Never or very rare”; “2 = Sometimes”, “3 = Often” and “4 = Very Often or always”. In scoring, for each student, the sum of the responses for the 20 items is obtained (range 20 to 80). This raw score is then converted to an “Anxiety Index” with values ranging from 25 to 100. According to Zung [31], an Anxiety Index < 45 indicates “Anxiety within normal range”, values in the range of 45–59 indicate “Minimal to moderate anxiety”, values in the range of 60–74 indicate “Marked to severe anxiety” and values ≥ 75 indicates “Most extreme anxiety’. The questionnaire was translated in local languages, where necessary, and was pilot tested among a group of university students in the respective countries. In pilot studies the Cronbach’s alpha values were more than 0.7.

### Coping strategies

The questionnaire used for evaluating students’ coping strategies was designed adapting questions from previous studies. In this study, the usage of four coping strategies were assessed: seeking social support, acceptance, mental disengagement, and humanitarian. Seeking social support refers to emotional or instrumental support received from family and friend that provide stress-related interpersonal aid [32]. An example of the questions: “*During Covid-19 and lockdown*, *I talked to someone about how I was feeling*”. Acceptance is the adaptation to unchangeable negative events by helping to maintain the individual’s psychological well-being and capacity to act [33]. An example of questions asked: “*About Covid-19 and lockdown*, *I learned to live with it*”. Mental disengagement involves directing attention and effort toward the goal of alleviating negative emotion by engaging in substitute activities to keep one’s mind from ongoing stressors [34]. An example of questions asked: “*To take my mind away from Covid-19 and lockdown*, *I watched TV*”. Humanitarian coping involves the initiatives taken by one in helping others in psychosocial despair. An example of questions asked: “*During Covid-19 and lockdown*, *I called/texted/videoed my friends to give them emotional support*.” This questionnaire was content validated by a panel of psychologist. The items were measured on a scale of 1 to 4: 1 = *never/rarely*, 2 = *sometimes*, 3 = *often*, and 4 = *very often/always*. The questionnaire was translated in local languages, where necessary. In pilot studies in the seven countries, Cronbach’s alpha values were more than 0.7 for the items in seek social support (4 items), acceptance (4 items), mental disengagement (2 items) and humanitarian (3 items) constructs, respectively. In the final analysis, for each coping strategy, the mean scores for the respective items were computed; higher scores implied higher level of use.

### Data analysis

In data analysis, the IBM SPSS Statistics for Windows, Version 24.0 (Armonk, NY). software was used. Quantitative variables summarized as means and standard deviations while qualitative variables were described as frequencies and percentages. In data analysis, t-test, ANOVA and MANOVA procedures were used. Level of significance was set as 0.05 for all tests.

## Results

In this cross-country survey, a total of 3,679 responses were received. The number of responses from each country, gender distribution and accommodation at the time of survey are shown in Table 1. Overall, there were more females than males in the sample. Most students from Malaysia, Saudi Arabia, Pakistan, India and Indonesia stayed in their family homes.

**Table 1:** Distribution of respondents in this study.

Country	Overall	Gender		Accommodation	
		Male	Female	Family Home	Rented Premises
Malaysia	983(26.7%)	330(33.6%)	653(66.4%)	850(86.5%)	133(13.5%)
Saudi Arabia	400(10.9%)	99(24.8%)	301(75.3%)	359(89.8%)	41(10.2%)
Pakistan	494(13.4%)	193(39.1%)	301(60.9%)	449(90.9%)	45(9.1%)
Bangladesh	474(12.9%)	293(61.8%)	181(32.8%)	75(15.8%)	399(84.2%)
China	559(15.2%)	333(59.6%)	226(40.4%)	309(55.3%)	250(44.7%)
India	364(9.9%)	147(40.4%)	217(59.6%)	304(83.5%)	60(16.5%)
Indonesia	405(11.0%)	124(30.6%)	281(69.4%)	348(85.9%)	57(14.1%)
Total	3679	1519(41.3%)	2160(58.7%)	2694(73.2%)	985(26.8%)

In this study, 2370(64.4%), 805(21.9%), 207(5.6%) and 297(8.1%) of the students experienced within normal range, mild to moderate, marked to severe and most extreme levels of anxiety, respectively. In view of the low frequencies, in the final analysis, the two levels of anxiety, “marked to severe” and “most extreme” were grouped together and named as “severe to extreme” level of anxiety. The level of anxiety experienced by the students by country is presented in Table 2 and the comparison of the level of anxiety between male and female in each country is shown in Table 3.

**Table 2:** Level of anxiety by country.

Country	Normal	Mild to Moderate	Severe to Extreme
*All counties*	*2370(64*.*4%)*	*805(21*.*9%)*	*504(13*.*7%)*
China	374(66.9%)	52(9.3%)	133(23.8%)
Bangladesh	183(38.6%)	192(40.5%)	99(20.9%)
Pakistan	200(58.7%)	125(25.3%)	79(16.0%)
Saudi Arabia	262(65.5%)	86(21.5%)	52(13.0%)
Indonesia	278(68.6%)	97(24.0%)	30(7.4%)
Malaysia	689(70.1%)	201(20.4%)	93(9.5%)
India	294(80.8%)	52(14.3%)	18(4.9%)

**Table 3:** Level of anxiety between male and female students by country.

Country	Gender	Anxiety level			Chi-square	p-value
		Normal	Minimal to Moderate	Severe to extreme		
*All Overall*	*F*	*1327(61*.*4%)*	*480(22*.*7%)*	*343(15*.*9%)*		
	*M*	*1043(68*.*7%)*	*315(20*.*7%)*	*161(10*.*6%)*		
Bangladesh	F	41(22.7%)	82(45.3%)	58(32.0%)	38.427	<0.001
	M	142(48.5%)	110(37.5%)	41(14.0%)		
China	F	135(59.7%)	17(7.5%)	74(32.7%)	16.983	<0.001
	M	239(71.8%)	35(10.5%)	59(17.7%)		
India	F	120(80.2%)	33(15.2%)	10(4.6%)	0.465	0.792
	M	191(81.6%)	19(12.9%)	8(5.4%)		
Indonesia	F	191(68.0%)	67(23.3%)	23(8.2%)	0.814	0.666
	M	87(70.2%)	30(24.2%)	7(5.6%)		
Malaysia	F	442(67.7%)	138(21.1%)	73(11.2%)	8.122	0.017
	M	247(74.8%	63(19.1%)	20(6.1%)		
Pakistan	F	158(52.5%)	82(27.2%)	61(20.3%)	15.010	<0.001
	M	132(67.4%)	43(22.3%)	18(9.3%)		
Saudi Arabia	F	186(61.78%)	71(23.6%)	44(14.6%)	7.465	0.024
	M	76(76.8%)	15(15.2%)	8(8.1%)		

There was a significant difference in the level of anxiety between students from different countries (Chi-square = 309.26, df = 12, p-value < 0.001). The proportion of severe to extreme level of anxiety was high among the students from China (23.8%) and Bangladesh (20.9%) and was low among the students from Indonesia (7.4%), Malaysia (9.5%) and India (1.9%).

As shown in Table 3, proportionately, more females experienced higher level of anxiety compared to the males. There were significant differences in levels of anxiety between the male and female students in Bangladesh, China, Malaysia, Pakistan and Saudi Arabia. In these five countries, the levels of anxiety were significantly higher among the female students in comparison to their male counterparts.

### Coping strategies used

The factor structure of the items in the respective coping strategies was good. The descriptive summaries for the four coping strategies of Seeking Social support, Acceptance, Mental Disengagement and Humanitarian by country and the comparison between male and female students within the countries are provided in Tables 4 and 5. The range for the scores is from 1 to 4. High scores indicate higher level of usage of the coping strategy.

**Table 4:** Descriptive summaries for the four coping strategies by country.

Country	Coping strategy			
	Seeking Social support	Acceptance	Mental Disengagement	Humanitarian
*All countries*	2.10 ± 0.83	2.62 ± 0.86	2.37 ± 0.68	2.25 ± 0.80
Bangladesh	2.04 ± 0.84	2.13 ± 0.84	2.12 ± 0.81	2.05 ± 0.86
China	2.28 ± 0.95	2.34 ± 0.92	2.55 ± 0.75	2.33 ± 0.90
India	2.03 ± 0.80	2.64 ± 0.87	2.20 ± 0.65	2.12 ± 0.73
Indonesia	2.17 ± 0.74	2.49 ± 0.87	2.20 ± 0.65	2.24 ± 0.73
Malaysia	2.08 ± 0.78	3.02 ± 0.72	2.55 ± 0.61	2.30 ± 0.71
Pakistan	1.98 ± 0.77	2.48 ± 0.79	2.07 ± 0.55	2.14 ± 0.71
Saudi Arabia	2.12 ± 0.87	2.94 ± 0.81	2.54 ± 0.63	2.53 ± 0.80
p-value	<0.001	<0.001	<0.001	<0.001

**Table 5:** Coping strategies used between male and female students by country.

Country	Gender	Coping strategy			
		Seeking Social support	Acceptance	Mental Disengagement	Humanitarian
*All countries*	F	2.17 ± 0.83	2.73 ± 0.82	2.40 ± 0.66	2.33 ± 0.80
	M	2.00 ± 0.80 (<0.001)	2.47 ± 0.90 (<0.001)	2.33 ± 0.71 (0.003)	2.15 ±0.78 (<0.001)
Bangladesh	F	2.28 ± 0.96	2.34 ± 0.95	2.39 ± 0.91	2.27 ± 1.00
	M	1.89 ± 0.73 (<0.001)	1.97 ± 0.71 (<0.001)	1.95 ± 0.69 (<0.001)	1.91 ± 0.73 (<0.001)
China	F	2.33 ± 1.03	2.42 ± 0.97	2.28 ± 0.88	2.36 ± 0.88
	M	2.25 ± 0.89 (0.365)	2.28 ± 0.88 (0.073)	2.65 ± 0.81 (0.009)	2.32 ± 0.83 (0.631)
India	F	2.16 ± 0.81	2.70 ± 0.83	2.19 ± 0.63	2.23 ± 0.74
	M	1.85 ± 0.75 (<0.001)	2.55 ± 0.93 (0.127)	2.20 ± 0.69 (0.893)	1.98 ± 0.71 (0.002)
Indonesia	F	2.22 ± 0.72	2.53 ± 0.69	2.31 ± 0.51	2.26 ± 0.70
	M	2.07 ± 0.76 (0.056)	2.40 ± 0.76 (0.074)	2.37 ± 0.57 (0.296)	2.19 ± 0.73 (0.402)
Malaysia	F	2.12 ± 0.78	3.02 ± 0.69	2.51 ± 0.57	2.34 ± 0.75
	M	2.00 ± 0.77 (0.024)	3.02 ± 0.78 (0.954)	2.64 ± 0.66 (0.001)	2.24 ± 0.75 (0.051)
Pakistan	F	2.06 ± 0.79	2.56 ± 0.74	2.08 ± 0.52	2.23 ± 0.70
	M	1.87 ± 0.73 (0.008)	2.35 ± 0.84 (0.004)	2.05 ± 0.59 (0.568)	2.00 ± 0.69 (0.001)
Saudi Arabia	F	2.18 ± 0.86	2.94 ± 0.79	2.53 ± 0.64	2.57 ± 0.79
	M	1.01 ± 0.86 (0.006)	2.92 ± 0.85 (0.837)	2.57 ± 0.58 (0.514)	2.42 ± 0.83 (0.128)

Values in parenthesis are the p-values.

As shown in Table 4, overall, the usage of the Acceptance Strategy was the highest and usage of Seeking Social Support strategy was the lowest. There were significant differences in all four coping strategies between students from different countries. Across the countries, the usage of Seeking Social Support strategies was more common among the students from China, and it was the least among the students from Pakistan. Students from Malaysia and Saudi Arabia used more Acceptance strategies compared to the students from Bangladesh. The usage of Mental Disengagement strategy was high among the students from China, Malaysia and Saudi Arabia compared to those from Bangladesh and Pakistan. In terms of Humanitarian coping, the usage was the highest among the students from Saudi Arabia compared to those from Bangladesh.

As shown in Table 5, in Bangladesh, the female students used all the four strategies more than the male students. In China, the usage of mental engagement among the male students was higher compared to their female counterpart. In India, the females used more adaptive strategies compared to the males. In Malaysia, the usage of seeking social support was higher among the females, while the usage of mental disengagement was higher among the male students. In Pakistan, the usage of seeking support, acceptance and humanitarian strategies were higher among the female students. In Saudi Arabia, the usage of seeking support strategy was higher among the female students.

## Discussion

This study examined the anxiety level among university students in seven Asian countries Based on the results, the proportion of severe to extreme level of anxiety among the university students was the highest in China (23.8%) and Bangladesh (20.9%) and was low in Indonesia (7.4%), Malaysia (9.5%) and India (1.9%). In the month of January, the disease spread like wildfire and the death toll skyrocketed in China. At that time, there was a lot of uncertainty on the causal agent and the disease spread mechanism. There was continuous coverage of people taken ill, insufficient hospital facilities and scenes of people dying were constantly flashed on the televisions and social media networks. Even though China declared that the peak of the outbreak of novel coronavirus disease in the country was over on 12^th^ March, it seems the grave experiences the students had was still lingering and haunting their minds beyond April 2020. This finding is similar to that Cao et al., [35]; Wang et al., [36]; and Zhang et al., [37].

Another reason for the high levels of anxiety among the students from China and Bangladesh could be their living arrangement during the time of the survey. A subsequent analysis showed a significant difference in the extent of severe to extreme level of anxiety among the students who stayed at home (10.1%) and those who stayed in rented premises (23.6%). In this study, 84.2% and 44.7% of the respondents from Bangladesh and China were staying in rented premises, away from their families, compared to less than 17% from the other countries. As suggested by Sundarasen et al. [38], students who stayed on their own are separated from their loved ones and the unexpected risk to their wellbeing and safety during this pandemic could have contributed to the feeling of loneliness and created unprecedented challenges from several angles.

The findings in this study also indicate that the female students from Bangladesh, China, Malaysia, Pakistan and Saudi Arabia experienced high level of anxiety compared to the male counterparts. This finding is similar to the study of Azad et al. [39] and Mirza and Jenkins [40]. Females usually convey feelings to a larger degree than males do, and this condition may have been intensified by the current pandemic. Studies suggest that female are less tolerant of uncertainty, and this pandemic is characterized by uncertainty; no one knows exactly what’s going to happen, and when or whether things are going to be back to normality.

Different people may use different coping strategies or combination of strategies as an effort to manage specific external and/or internal demands that are appraised as taxing or exceeding the resources of the person. In this study we assessed four type of coping strategies: Seeking Social Support, Acceptance, Mental Disengagement and Humanitarian strategies. Overall, the usage of Acceptance strategy was the highest and usage of Seeking Social Support strategy was the lowest. There were significant differences in all four coping strategies between the students from different countries. The usage of Seeking Social Support strategies (like “*I talk to someone about how I feel*”) was more common among the students from China and it was the least common among the students from Pakistan. This could be due to the disparity in social media connectivity in the two countries. Students from Malaysia and Saudi Arabia used more Acceptance strategies (like “*I learn to live with it*”) compared to the students from Bangladesh. This could be due to cultural differences. The usage of Mental Disengagement strategy (like “*To take my mind away I watch TV*”) was high among the students from China, Malaysia and Saudi Arabia compared to those from Bangladesh and Pakistan. This could be due the differences in the availability of facilities and programs. In terms of Humanitarian coping (like “*I call/text/video my friends to give them emotional support*”), the usage was the higher among the students from Saudi Arabia compared to those from Bangladesh. Across countries, the female students used more social support strategy, while their male counterpart used more mental disengagement strategy.

During the COVID-19 pandemic, countries across the globe took various measures to control the spread of the disease [41]. Lockdown was one of the mitigation actions taken by most countries [42]. During the lockdown period most of the economic activities came to a standstill, thus contributing to huge financial dilemma [43]. Places of public gatherings like places of worship, sport arenas, shopping complexes and academic institution were closed. Universities had to switch to virtual mode of teaching, literally overnight, and this has left many students clueless of what was happening [38].

Based on the descriptive feedback from the students in the survey, monetary constrictions, remote online learning, ambiguity in terms of their academic performance, completion of study and future career prospects were the main stressors. Financially, the students were disturbed with their capacity to handle their educational financial obligations due to loss of income by family breadwinners and lack of openings to self-finance their studies. In several countries in the Asian region, university students provide private tuitions to finance their studies [19]. Disruption in this source of income to finance their studies and to financially assist their family due to the lockdown, could have increased the levels of anxiety and depression among the university students. Although restrictions and attempts of social distancing are saving lives, there is also a growing financial damage as some firms are laying off their employees.

Another main contributing factor to anxiety and stress is the move to remote online teaching and learning [38]. Students are confronted with arduous challenges in terms of technological infrastructure such as lack of internet facility, weak internet connectivity and frequent power outages [44] specifically those in Pakistan, Bangladesh, and Indonesia. These short comings limit students’ attendance of online classes. Students are also undergoing tremendous anxiety with online exams with many just not able to take the exams.

In the sudden move to online remote teachings, most educational institutions still used the existing curricula and learning outcomes which suited the face-to-face instructions [45]. This amplified the stress and anxiety level as students are unduly hampered with course work and assignments. Instructors, on the other hand are futile in realizing the tough moments students are enduring mentally and emotionally. The students are torn between remote learning and separation from friends and loved ones and this had created unwarranted frustration, annoyance, detestation and ultimately, anxiety. Graduating students were extremely distraught as they were destitute in their career plans.

As with other studies employing questionnaires, the limitation of this study includes students’ honesty in answering the questions, which could not be verified. These data were collected during the months of April and May 2020, when the university residences were forced to be closes and most students had returned to their family homes. There is a possibility that if the data were obtained at the peak time of COVID-19, January and February 2020 in China and mid-March 2020 in other countries, the levels of anxiety of the students may have been different. That being said, it was not feasible as we had to wait for the ethical clearance of the authorities concerned and formalizing the collaborations.

## Conclusions

About one-third of the students in the study experienced some level of anxiety during the COVID-19 pandemic and lockdown period. Acceptance strategy was the highest, while Seeking Social Support strategy was the lowest used in mitigating anxiety. Female students used more social support strategy; the male students used more mental disengagement strategy. The students are not prepared to the “new norms”. Recommendations include the need for guidance, counselling and assistance for the students to pull through this testing times. Establishing and sustaining connections with the students is important for their psychological and social well-being and is among the defining characteristics of university experience. Academic institutions must reevaluate existing curricula, learning outcomes, and evaluation strategies for effective online mode of delivery.

## Supporting information

S1 File Data in SPSS. (SAV)

## References

[1] Matias T, Dominski FH, Marks DF. Human needs in COVID-19 isolation. J Health Psychol. 2020;1–12. doi: 10.1177/1359105320925149 32375564

[2] Tull MT, Edmonds KA, Scamaldo KM, Richmond JR, Rose JP, Gratz KL. Psychological Outcomes Associated with Stay-at-Home Orders and the Perceived Impact of COVID-19 on Daily Life. Psychiatry Res [Internet]. 2020;289(April):113098. Available from: 10.1016/j.psychres.2020.113098 32434092 PMC7252159

[3] Brooks SK, Webster RK, Smith LE, Woodland L, Wessely S, Greenberg N, et al. The psychological impact of quarantine and how to reduce it: rapid review of the evidence. Lancet [Internet]. 2020;395(10227):912–20. Available from: 10.1016/S0140-6736(20)30460-8 32112714 PMC7158942

[4] Liang L, Ren H, Cao R, Hu Y, Qin Z, Li C, et al. The Effect of COVID-19 on Youth Mental Health. Psychiatr Q. 2020;(1163). doi: 10.1007/s11126-020-09744-3 32319041 PMC7173777

[5] Abdullah JM, Wan Ismail WFN, Mohamad I, Ab Razak A, Harun A, Musa KI, et al. A critical appraisal of COVID-19 in Malaysia and beyond. Malaysian J Med Sci. 2020;27(2):1–9. doi: 10.21315/mjms2020.27.2.1 32308536 PMC7153689

[6] Asmundson GJG, Taylor S. Coronaphobia: Fear and the 2019-nCoV outbreak. J Anxiety Disord [Internet]. 2020;70:102196. Available from: http://www.sciencedirect.com/science/article/pii/S0887618520300104 32078967 10.1016/j.janxdis.2020.102196 PMC7134790

[7] Courtet P, Olié E, Debien C, Vaiva G. Keep Socially (but Not Physically) Connected and Carry on: Preventing Suicide in the Age of COVID-19. J Clin Psychiatry [Internet]. 2020;81 (3)(April 14). Available from: https://www.psychiatrist.com/JCP/article/Pages/preventing-suicide-in-the-age-of-covid.aspx 32297718 10.4088/JCP.20com13370

[8] Reger MA, Stanley IH, Joiner TE. Suicide Mortality and Coronavirus Disease 2019—A Perfect Storm? JAMA Psychiatry [Internet]. 2020 Apr 10; Available from: 10.1001/jamapsychiatry.2020.1060 32275300

[9] Carvalho PM de M, Moreira MM, de Oliveira MNA, Landim JMM, Neto MLR. The psychiatric impact of the novel coronavirus outbreak. Psychiatry Res [Internet]. 2020 Feb 28;286:112902. Available from: https://pubmed.ncbi.nlm.nih.gov/32146248 32146248 10.1016/j.psychres.2020.112902 PMC7133679

[10] Tang W, Hu T, Hu B, Jin C, Wang G, Xie C, et al. Prevalence and correlates of PTSD and depressive symptoms one month after the outbreak of the COVID-19 epidemic in a sample of home-quarantined Chinese university students. Vol. 274, Journal of Affective Disorders. 2020. p. 1–7. 10.1016/j.jad.2020.05.009 PMC7217769 32405111

[11] Chang J, Yuan Y, Wang D. Mental health status and its influencing factors among college students during the epidemic of COVID-19. J South Med Univ. 2020;40(2):171–6. doi: 10.12122/j.issn.1673-4254.2020.02.06 32376528 PMC7086131

[12] Liu S, Liu Y, Liu Y. Somatic symptoms and concern regarding COVID-19 among Chinese college and primary school students: A cross-sectional survey. Psychiatry Res [Internet]. 2020;289(April):113070. Available from: 10.1016/j.psychres.2020.113070 32422501 PMC7227526

[13] Bayram N, Bilgel N. The prevalence and socio-demographic correlations of depression, anxiety and stress among a group of university students. Soc Psychiatry Psychiatr Epidemiol [Internet]. 2008;43(8):667–72. Available from: 10.1007/s00127-008-0345-x 18398558

[14] Auerbach RP, Alonso J, Axinn WG, Cuijpers P, Ebert DD, Green JG, et al. Mental disorders among college students in the World Health Organization World Mental Health Surveys. Psychol Med [Internet]. 2016/08/03. 2016;46(14):2955–70. Available from: https://www.cambridge.org/core/article/mental-disorders-among-college-students-in-the-world-health-organization-world-mental-health-surveys/34942DEAFC35899349114B73E84FB080 27484622 10.1017/S0033291716001665 PMC5129654

[15] Bruffaerts R, Mortier P, Kiekens G, Auerbach RP, Cuijpers P, Demyttenaere K, et al. Mental health problems in college freshmen: Prevalence and academic functioning. J Affect Disord [Internet]. 2018;225:97–103. Available from: http://www.sciencedirect.com/science/article/pii/S0165032716324545 28802728 10.1016/j.jad.2017.07.044 PMC5846318

[16] Bao Y, Sun Y, Meng S, Shi J, Lu L. 2019-nCoV epidemic: address mental health care to empower society. Lancet [Internet]. 2020 Feb 22;395(10224):e37–8. Available from: 10.1016/S0140-6736(20)30309-3 32043982 PMC7133594

[17] Sher L. The impact of the COVID-19 pandemic on suicide rates. QJM: An International Journal of Medicine. 2020 Oct;113(10):707–12. doi: 10.1093/qjmed/hcaa202 32539153 PMC7313777

[18] Odriozola-González P, Planchuelo-Gómez Á, Irurtia MJ, de Luis-García R. Psychological effects of the COVID-19 outbreak and lockdown among students and workers of a Spanish university. Psychiatry Res [Internet]. 2020;290(May):113108. Available from: 10.1016/j.psychres.2020.113108 32450409 PMC7236679

[19] Serafini G, Gonda X, Canepa G, Pompili M, Rihmer Z, Amore M, et al. Extreme sensory processing patterns show a complex association with depression, and impulsivity, alexithymia, and hopelessness. Journal of affective disorders. 2017 Mar 1;210:249–57. doi: 10.1016/j.jad.2016.12.019 28064114

[20] Islam MA, Barna SD, Raihan H, Khan MNA, Hossain MT. Depression and anxiety among university students during the COVID-19 pandemic in Bangladesh: A web-based cross-sectional survey. PLoS One [Internet]. 2020 Aug 26;15(8):e0238162. Available from: 10.1371/journal.pone.0238162 32845928 PMC7449469

[21] Lazarus RS, Folkman S. Stress, appraisal, and coping. New York: Springer Publication; 1984.

[22] Carver C, Scheier M, Weintraub J. Assessing Coping Strategies: A Theoretically Based Approach, Journal of Personality and Social Psychology. J Pers Soc Psychol. 1989;56(2):267–83. doi: 10.1037//0022-3514.56.2.267 2926629

[23] Penley JA, Tomaka J, Wiebe JS. The Association of Coping to Physical and Psychological Health Outcomes: A Meta-Analytic Review. J Behav Med. 2002;25(6):551–603. doi: 10.1023/a:1020641400589 12462958

[24] Mahmoud JSR, Staten RT, Lennie TA, Hall LA. The Relationships of Coping, Negative Thinking, Life Satisfaction, Social Support, and Selected Demographics With Anxiety of Young Adult College Students. J Child Adolesc Psychiatr Nurs. 2015;28(2):97–108. doi: 10.1111/jcap.12109 25939686

[25] Blanchard-Fields F. Everyday Problem Solving and Emotion: An Adult Developmental Perspective. Curr Dir Psychol Sci [Internet]. 2007 Feb 1;16(1):26–31. Available from: 10.1111/j.1467-8721.2007.00469.x

[26] Folkman S. Stress, Health, and Coping: Synthesis, Commentary, and Future Directions. Oxford Handb Stress Heal Coping. 2012;d:1–15.

[27] Tein JY, Sandler IN, Zautra AJ. Stressful life events, psychological distress, coping, and parenting of divorced mothers: A longitudinal study. J Fam Psychol. 2000;14(1):27–41. doi: 10.1037//0893-3200.14.1.27 10740680

[28] Main A, Zhou Q, Ma Y, Luecken LJ, Liu X. Relations of sars-related stressors and coping to chinese college students’ psychological adjustment during the 2003 beijing sars epidemic. J Couns Psychol. 2011;58(3):410–23. doi: 10.1037/a0023632 21574694

[29] Beck AT, Clark DA. An information processing model of anxiety: Automatic and strategic processes. Behav Res Ther. 1997;35(1):49–58. doi: 10.1016/s0005-7967(96)00069-1 9009043

[30] Deasy C, Coughlan B, Pironom J, Jourdan D, Mannix-McNamara P. Psychological distress and coping amongst higher education students: A Mixed method enquiry. PLoS One. 2014;9(12):1–23. doi: 10.1371/journal.pone.0115193 25506825 PMC4266678

[31] Kelly MM, Tyrka AR, Price LP, Carpenter LL. Sex Differences in the Use of Coping Strategies: Depress Anxiety. 2008;25(10):839–46. doi: 10.1002/da.20341 17603810 PMC4469465

[32] Zung WWK. A Rating Instrument For Anxiety Disorders. Psychosomatics [Internet]. 1971;12(6):371–9. Available from: http://www.sciencedirect.com/science/article/pii/S0033318271714790 5172928 10.1016/S0033-3182(71)71479-0

[33] Dumont M, Provost MA. Resilience in Adolescents: Protective Role of Social Support, Coping Strategies, Self-Esteem, and Social Activities on Experience of Stress and Depression. J Youth Adolesc [Internet]. 1999;28(3):343–63. Available from: 10.1023/A:1021637011732

[34] Nakamura YM, Orth U. Acceptance as a coping reaction: Adaptive or not? Swiss J Psychol. 2005;64(4):281–92.

[35] Traeger L. Distraction (Coping Strategy) BT—Encyclopedia of Behavioral Medicine. In: Gellman MD, Turner JR, editors. New York, NY: Springer New York; 2013. p. 610–1. 10.1007/978-1-4419-1005-9_179

[36] Cao W, Fang Z, Hou G, Han M, Xu X, Dong J, et al. The psychological impact of the COVID-19 epidemic on college students in China. Vol. 287, Psychiatry Research. 2020. doi: 10.1016/j.psychres.2020.112934 32229390 PMC7102633

[37] Wang Y, Zhao X, Feng Q, Liu L, Yao Y, Shi J. Psychological assistance during the coronavirus disease 2019 outbreak in China. J Health Psychol. 2020;25(6):733–7. doi: 10.1177/1359105320919177 32301628

[38] Sundarasen S, Chinna K, Kamaludin K, Nurunnabi M, Baloch GM, Khoshaim HB, et al. Psychological impact of covid-19 and lockdown among university students in malaysia: Implications and policy recommendations. Int J Environ Res Public Health. 2020;17(17):1–13. doi: 10.3390/ijerph17176206 32867024 PMC7504527

[39] Azad N, Shahid A, Abbas N, Shaheen A, Munir N. Anxiety And Depression In Medical Students Of A Private Medical College. J Ayub Med Coll Abbottabad. 2017;29(1):123–7. 28712190

[40] Mirza I, Jenkins R. Risk factors, prevalence, and treatment of anxiety and depressive disorders in Pakistan: systematic review. BMJ [Internet]. 2004 Apr 1;328(7443):794. Available from: http://www.bmj.com/content/328/7443/794.abstract 15070634 10.1136/bmj.328.7443.794 PMC383372

[41] Wilder-Smith A, Freedman D.O. Isolation, quarantine, social distancing and community containment: pivotal role for old-style public health measures in the novel coronavirus (2019-nCoV) outbreak, Journal of Travel Medicine. 2020; 27(2), 10.1093/jtm/taaa020 32052841 PMC7107565

[42] Askitas N, Tatsiramos K, Verheyden B. Lockdown strategies, mobility patterns and Covid-19. Covid Economics Vetted and Real-Time Papers. 2020; (23): 263–304

[43] Sibley et al. Effects of the COVID-19 Pandemic and Nationwide Lockdown on Trust, Attitudes Toward Government, and Well-Being. American Psychologist. 2020; 75(5): 618–630 10.1037/amp0000662 32496074

[44] Adnan M, Anwar K Online learning amid the COVID-19 pandemic: Students’ perspectives Journal of Pedagogical Sociology and Psychology 2020; 2(1);45–51 10.33902/JPSP.2020261309

[45] Zhang W, Wang Y, Yang L, Wang C. Suspending Classes Without Stopping Learning: China’s Education Emergency Management Policy in the COVID-19 Outbreak. J Risk Financ Manag. 2020;13(3):55.

