# Correction: Psychological impact of COVID-19 and lock down measures: An online cross-sectional multicounty study on Asian university students

**DOI:** 10.1371/journal.pone.0306485

**Published:** 2024-06-27

**Authors:** Karuthan Chinna, Sheela Sundarasen, Heba Bakr Khoshaim, Kamilah Kamaludin, Mohammad Nurunnabi, Gul Mohammad Baloch, Syed Far Abid Hossain, Areej Sukayt, Nevi Danila, Usha Rajagopalan, Ramesh Kumar, Zahid Memon

The ninth author’s name is spelled incorrectly. The correct name is: Nevi Danila.

The affiliation for the ninth author is incorrect. The correct affiliation is not indicated. Nevi Danila is not affiliated with #2 but with: Finance Department, Prince Sultan University.
